# Applicability and predictive validity of the global leadership initiative on malnutrition criteria for older patients with sepsis according to different muscle mass assessment methods

**DOI:** 10.1016/j.jnha.2025.100685

**Published:** 2025-09-19

**Authors:** Na Shang, Qiujing Li, Haijing Zhou, Xiangqun Zhang, Shubin Guo, Xue Mei

**Affiliations:** a Emergency Medicine Clinical Research Center, Beijing Chaoyang Hospital, Capital Medical University, Beijing 100020, China; b Department of Emergency Medicine, Beijing Shijitan Hospital, Capital Medical University, Beijing 100038, China

**Keywords:** Malnutrition, Global leadership initiative on malnutrition, Sepsis, Older adults

## Abstract

**Objectives:**

To evaluate the applicability of the Global Leadership Initiative on Malnutrition (GLIM) criteria in older patients with sepsis and to compare the predictive validity for 28-day mortality of different muscle mass assessment methods in the emergency department.

**Design:**

Prospective cohort study.

**Setting:**

Emergency department.

**Patients:**

Older patients (≥65 years) with sepsis.

**Measurements:**

Muscle mass was assessed using three methods: (1) the skeletal muscle index at the third lumbar vertebra (L3) on computed tomography (CT) scans; (2) calf circumference (CC), and (3) mid-upper-arm circumference (MAC). Cox regression analysis was performed to assess the association between the GLIM criteria and 28-day all-cause mortality. Additionally, the C-statistic, net reclassification improvement (NRI), and integrated discrimination improvement (IDI) were used to evaluate the predictive validity of the three instruments. Survival curves were assessed using the Kaplan–Meier method and compared using the log-rank test.

**Results:**

A total of 598 patients with sepsis were included. The prevalence of malnutrition according to GLIM-CT, GLIM-CC, and GLIM-MAC was 53.3%, 63.0%, and 40.8%, respectively. Cox regression analysis revealed that the GLIM criteria were independent risk factors for all-cause 28-day mortality. Incorporation of GLIM-CT, GLIM-CC, or GLIM-MAC into a base model significantly improved the C-statistic. The model including GLIM-CT had the highest C-statistic, improving the C-statistic of the base model from 0.780 (95% confidence interval [CI]: 0.741−0.819) to 0.823 (95% CI: 0.789−0.857). This improvement in risk prediction was also confirmed via category-free NRI and IDI, suggesting that GLIM-CT had the best performance. Kaplan–Meier survival analysis showed that patients with malnutrition defined according to the GLIM criteria had a greater probability of 28-day mortality (log-rank, *P* < 0.001).

**Conclusion:**

Malnutrition, defined via any of the three methods, was predictive of 28-day mortality among older patients with sepsis in the emergency department. GLIM-CT had the best predictive validity.

## 1 Introduction

According to Sepsis-3.0 criteria, sepsis is defined as a life-threatening organ dysfunction caused by a dysregulated host response to infection [1]. Older patients account for the majority of cases (60%–85%) [2], with in-hospital mortality ranging from 30% to 60% among patients over 65 years of age and increasing to 40%–80% in those aged 80 years and above [3]. Sepsis often first takes place at the emergency department [4], and as populations age, it poses a substantial burden on healthcare systems. Several risk factors contribute to sepsis-related mortality and morbidity among older adults, including immunosenescence, malnutrition, comorbidities, sarcopenia, frailty, and cognitive impairment [5].

Among the abovementioned risk factors, malnutrition is a major health problem worldwide, especially in older adults [6]. Approximately a quarter of older adults (aged ≧65 years) are malnourished or at risk of malnutrition [7]. The reported prevalence of malnutrition among older patients with sepsis varies considerably between studies owing to the different nutritional assessment tools used. Recent studies have demonstrated its notable impact on adverse clinical outcomes of sepsis among older adults [[8], [9], [10]]. In 2018, the Global Leadership Initiative on Malnutrition (GLIM)^1^ proposed a consensus scheme for the diagnosis of malnutrition in clinical settings (Table 1). A diagnosis requires the presence of at least one phenotypic criterion (weight loss, a low body mass index [BMI], or a reduced muscle mass) and one etiologic criterion (reduced food intake or assimilation or disease burden/inflammation) [11].

**Table 1:** Phenotypic and etiologic criteria for the diagnosis of malnutrition within this study.

Phenotypic criteria			Etiologic criteria	
Weight loss	Low BMI(kg/m^2^)	Reduced muscle mass	Reduced food intake or assimilation	Disease burden/inflammation
>5% within past 6 months, or >10% beyond 6 months	<18.5 if <70 years, or <20 if >70 years	L3 SMI: <28.6 cm^2^ in women and <37.9 cm^2^ in men; CC: ＜32 cm in women and ＜33 cm in men; MAC: ＜21 cm;	≤50% of the intake with respect to the usual in the last week or any reduction >2 weeks or the presence of diseases that alter the absorption of food	All patients with sepsis based on acute disease

Abbreviations: L3 SMI, skeletal mass index of the third lumbar vertebra; CC, calf circumference; MAC, mid-upper arm circumference.

Although reduced muscle mass is one of the GLIM phenotypic criteria, no unified standard exists for its measurement and definition in clinical practice. The GLIM recommends use of the fat-free mass index, measured using dual-energy absorptiometry (DXA), bioelectrical impedance analysis (BIA), computed tomography (CT), or magnetic resonance imaging (MRI). When these tools are unavailable, standard anthropometric measures such as the mid-upper-arm circumference (MAC) or calf circumference (CC) can also be used. Functional assessments such as hand-grip strength (HGS) may serve as supportive proxies. Each assessment tool has limitations and advantages, and whether these tools accurate reflect reduced muscle mass and can be used to further evaluate malnutrition status is unclear, especially for older patients with sepsis in emergency settings.

Therefore, the purpose of this study was to explore the application of malnutrition, as defined by the GLIM criteria, according to different muscle mass measurement tools among older patients with sepsis in the emergency department, and to compare their predictive validity for 28-day mortality.

## 2 Material and methods

### 2.1 Population

This prospective, observational cohort study was conducted in a tertiary-care, university-affiliated hospital in Beijing. Consecutive patients aged ≥65 years who were admitted to the emergency department from January 2022 to November 2022 were eligible for this study. During the study period, only patients without coronavirus disease 2019 were admitted to our hospital, whereas those who tested positive were transferred to government-designated hospitals. This study was approved by the institutional review board of Beijing Chao-Yang Hospital affiliated to Capital Medical University (approval number: 2022-ke-430, August 1, 2022) and conducted in accordance with the amended Declaration of Helsinki. This study was registered at www.chictr.org.cn (registration number ChiCTR2300070377). Informed consent was obtained from each patient or their next of kin before enrolment in the study. All procedures were performed in compliance with relevant laws and institutional guidelines.

The inclusion criteria were as follows: **(**1) age ≥65 years; (2) diagnosed with sepsis according to Sepsis-3.0 criteria, that is, a suspected or confirmed infection plus an acute increase in the sequential organ failure assessment (SOFA) score (≥2); and (3) abdominal CT performed within 24 h of emergency department admission.

The exclusion criteria were as follows: (1) discharged or transferred within 24 h of admission; (2) pre-existing or acute peripheral neuromuscular diseases; (3) CT imaging data that did not meet the quality checks; (4) incomplete clinical information; (5) radiotherapy and chemotherapy administered for tumors; (6) cachexia; (7) severe edema of the lower limbs.

### 2.2 GLIM diagnostic scheme of malnutrition

#### 2.2.1 Risk Screening for malnutrition using Nutritional Risk Screening 2002

The first step in the assessment of malnutrition was risk screening to identify those who were at risk of malnutrition by using the Nutritional Risk Screening 2002 (NRS2002). Patients with a score ≥3 were deemed to have a high risk of malnutrition [12]. Such patients were referred for further nutritional assessment, as described below.

#### 2.2.2 Diagnostic assessment criteria of malnutrition

##### 2.2.2.1 Phenotypic criteria

Weight loss (%): Involuntary weight loss was defined as >5% weight loss within the past 6 months or >10% weight loss over a longer period.

Low BMI (kg/m^2^): As participants in this study were Asian, a low BMI was considered as <18.5 kg/m^2^ for patients <70 years old and <20 kg/m^2^ for patients >70 years old according to European Society for Clinical Nutrition and Metabolism (ESPEN) recommendations [11].

Reduced muscle mass: Recommendations for measurement methods for muscle mass include DXA, BIA, CT, and MRI. When these techniques are not available, anthropometric measures, such as the MAC or CC may be used. In this study, we applied the following three methods to evaluate muscle loss and the standard operating procedures were as follows.

1) CT: Plain abdominal CT scans were performed within 24 h of admission, and images retrieved from the institutional picture archiving and communication system were analyzed using AW Volume Share 7 (GE HealthCare, Chicago, IL, USA). The built-in CT histogram software “X Section” on the workstation was used to manually delineate regions of interest at the midpoint of the L3 region, and the skeletal mass area (SMA) was automatically calculated by the software. The CT threshold for the L3 SMA was set to −29 to 150 Hounsfield units [13]. The skeletal mass index (SMI) was calculated as SMA (cm^2^) divided by the patient’s height squared (m^2^) [14]. This procedure was conducted by two trained emergency physicians, and the average of the two values was used for analysis. The cut-off values for muscle mass reduction (GLIM-CT) were <28.6 cm^2^ for women and <37.9 cm^2^ for men, based on thresholds in a multi-center CT study in China [15].
2) CC: With the patient in the supine position with their feet spread shoulder-width apart, non-elastic tape was used to measure the maximum circumference of both calves. We used BMI-adjusted CC values for patients outside the normal BMI range (18–24.9 kg/m^2^). The adjusted CC is calculated by adding 4 cm (for a BMI < 18.5) or subtracting 3, 7, or 12 cm (for a BMI of 25−29 kg/m^2^, 30−39 kg/m^2^, or ≥40 kg/m^2^, respectively) from the original CC measurement [16]. The cut-off points for GLIM−CC were <32 cm for females and <33 cm for males according to the guidance for assessment of the muscle mass phenotypic criterion for GLIM [17].
3) MAC: Patients were asked to raise their arms to shoulder level, with the elbow bent 90 °. In this position, patients were asked to flex their biceps, and the maximum circumference of the biceps was measured. The average of two measurements was calculated and used for analyses. The GLIM-MAC threshold was <21 cm, as recommended in previous studies [18,19].

##### 2.2.2.2 Etiologic criteria

As all patients in this study had sepsis, all met this criterion because they were diagnosed with acute disease.

#### 2.2.3 Diagnosis of malnutrition according to GLIM criteria

Weight loss, low BMI, and reduced muscle mass are classified as phenotypic criteria, while reduced food intake/assimilation and disease burden/inflammatory response are categorized as etiologic criteria (Table 1). The threshold values for diagnosis of malnutrition are shown in Table 1. For the diagnosis of malnutrition using GLIM, at least one phenotypic criterion and one etiologic criterion was required.

### 2.3 Study protocol

Patient characteristics, including age, gender, BMI, chronic diseases (such as hypertension, diabetes mellitus, coronary artery disease, cerebrovascular disease, chronic kidney disease, and chronic pulmonary disease), sites of infection (e.g., the lungs, abdomen, and urinary tract), and laboratory indicators were collected upon admission. The Charlson Comorbidity Index (CCI) was calculated for the chronic disease burden, and the SOFA score was calculated to assess disease severity. All data were collected within 24 h of admission.

### 2.4 Outcome measures

The outcome measure was 28-day all-cause mortality, which was ascertained from the patients’ electronic medical records or via telephonic follow-up.

### 2.5 Statistical analysis

Descriptive statistics are reported as the mean (standard deviation) for normally distributed variables, median (lower quartile [Q_L_], upper quartile [Q_U_]) for non-normally distributed variables, and counts (percentages) for categorical variables. Differences in characteristics between the groups were evaluated using chi-square tests for categorical variables. Cohen’s kappa coefficient was calculated to examine the agreement among GLIM-CT, GLIM-CC, and GLIM-MAC. Cox proportional-hazards models and Kaplan–Meier curves were used for survival analysis. Hazard ratios (HRs) and 95% confidence intervals (CIs) were calculated. C-statistics, category-free net reclassification improvement (NRI), and integrated discrimination improvement (IDI) were calculated to investigate the predictive ability of the three approaches. The log-rank test was used to compare Kaplan–Meier survival curves based on malnutrition as defined by the GLIM. All statistical analyses were performed using IBM SPSS Statistics for Windows (version 26.0; IBM Corp., Armonk, NY, USA) and GraphPad (version 8.4.0; GraphPad Software Inc., La Jolla, CA, USA). All *P*-values were two-tailed, and *P* < 0.05 was considered significant.

### 2.6 Sample size

Sample size calculation has not been explicitly conducted before initiating this study. Nevertheless, previous studies have reported a close association between malnutrition and mortality. Among critically ill patients, the mortality rate in the malnourished group ranges from 37.8% to 65%, while that in the non-malnourished group ranges from 10.6% to 28% [20,21]. Based on the clinically significant findings from prior studies and the following sample size calculation formula [22], the sample size included in this study can detect such an association with a significance level of 5% and a statistical power of 80%.

$$n=\frac{{\left({\text{Z}}_{1-\alpha /2}\sqrt{2\text{p}(1-\text{p})}+{\text{Z}}_{1-\beta }\sqrt{{\text{p}}_{1}\left(1-{\text{p}}_{1}\right)+{\text{p}}_{2}(1-{\text{p}}_{2})}\right)}^{2}}{{\left({\text{p}}_{1}-{\text{p}}_{2}\right)}^{2}}$$

Notes: n represents the required sample size per group, p_1_ and p_2_ represent the mortality rates of patients in the malnourished group and the non-malnourished group, respectively. p refers to the pooled rate of the two groups.

## 3 Results

### 3.1 Baseline characteristics

In this study, 598 older participants were included, of whom 329 (55.0%) were men. The median (interquartile range) age was 76.0 (16.0) years, and the BMI was 23.0 (5.1) kg/m^2^. The baseline data are summarized in Table 2. The sites of infection at admission were the lungs (42.0%), abdomen (39.3%), urinary tract (16.7%), and other sites (2.0%). Firstly, among these older patients with sepsis, 428 patients were identified as being at high risk of malnutrition screening with the NRS 2002 score. Subsequently, the numbers of malnourished patients as defined by GLIM-CT, GLIM-CC, and GLIM-MAC were 319, 377, and 244 respectively, with the corresponding prevalence of malnutrition being 53.3%, 63.0%, and 40.8%. The consistency of these three methods was relatively good, the highest agreement being between GLIM-CT and GLIM-MAC (Cohen’s kappa coefficient = 0.706, *P* < 0.001).

**Table 2:** Baseline characteristics of participants (N = 598).

Parameter	Total (n = 598)	GLIM-CT (n = 319)	GLIM-CC (n = 377)	GLIM-MAC (n = 244)
General characteristics				
Age, year, median (Q_L_, Q_U_)	76 (69, 85)	80 (72, 86)	80 (72, 86)	79 (72, 85)
Male, n (%)	329 (55.2)	173 (49.9)	174 (50.1)	174 (50.1)
BMI, kg/m^2^, mean (SD)	23.0 (5.1)	21.5 (4.5)	22.0 (5.0)	24.2 (4.2)
Commodities (n,%)				
Hypertension (n,%)	332 (55.5)	177 (55.5)	215 (57.0)	128 (52.5)
Diabetes mellitus (n,%)	220 (36.8)	108 (33.9)	131 (34.7)	78 (32.0)
Coronary artery disease (n,%)	188 (31.4)	94 (29.5)	117 (31.0)	78 (32.0)
Chronic kidney disease (n,%)	101 (16.9)	54 (16.9)	64 (17.0)	37 (15.2)
Cerebrovascular disease (n,%)	171 (28.6)	103 (32.3)	121 (32.1)	79 (32.4)
Chronic pulmonary disease (n,%)	68 (11.4)	39 (12.2)	49 (13.0)	30 (12.3)
Sites of Infection, n (%)				
Lung	251 (42.0)	158 (49.5)	183 (48.5)	121 (49.6)
Intra-abdominal infection	235 (39.3)	107 (33.5)	126 (33.4)	85 (34.8)
Urinary infection	100 (16.7)	46 (14.4)	60 (15.9)	32 (13.1)
Others	12 (2.0)	8 (2.5)	8 (2.1)	6 (2.5)
Laboratory indicators				
White blood cell, ×10^9^/L (Q_L_, Q_U_)	10.5 (7.2, 14.6)	10.6 (7.4, 15.4)	10.7 (7.5, 14.6)	10.2 (7.3, 15.3)
Hemoglobin, g/L, median (Q_L_, Q_U_)	11 7 (97, 136)	116 (93, 134)	116 (93, 135)	114 (91, 132)
Platelet, ×10^9^/L (Q_L_, Q_U_)	189 (137, 252)	206 (140, 272)	196 (138, 265)	201 (141, 275)
Serum albumin, g/dl (Q_L_, Q_U_)	36.0 (31.3, 40.5)	34.7 (30.7, 38.5)	35.0 (30.9, 39.4)	34.3 (30.7, 38.5)
Scores				
CCI, median (Q_L_, Q_U_)	5 (4, 7)	6 (4, 6)	6 (5, 7)	6 (5, 7)
SOFA, median (Q_L_, Q_U_)	4 (2, 7)	5 (3, 7)	5 (3, 7)	5 (3, 7)

Abbreviations: Q_L_, lower quartile; Q_U_, upper quartile; SD, standard deviation; BMI, body mass index; PCT, procalcitonin; CCI, Charlson comorbidity index; SOFA, Sequential Organ Failure Assessment.

### 3.2 Predictive validity of GLIM criteria

**Fig. 1:**
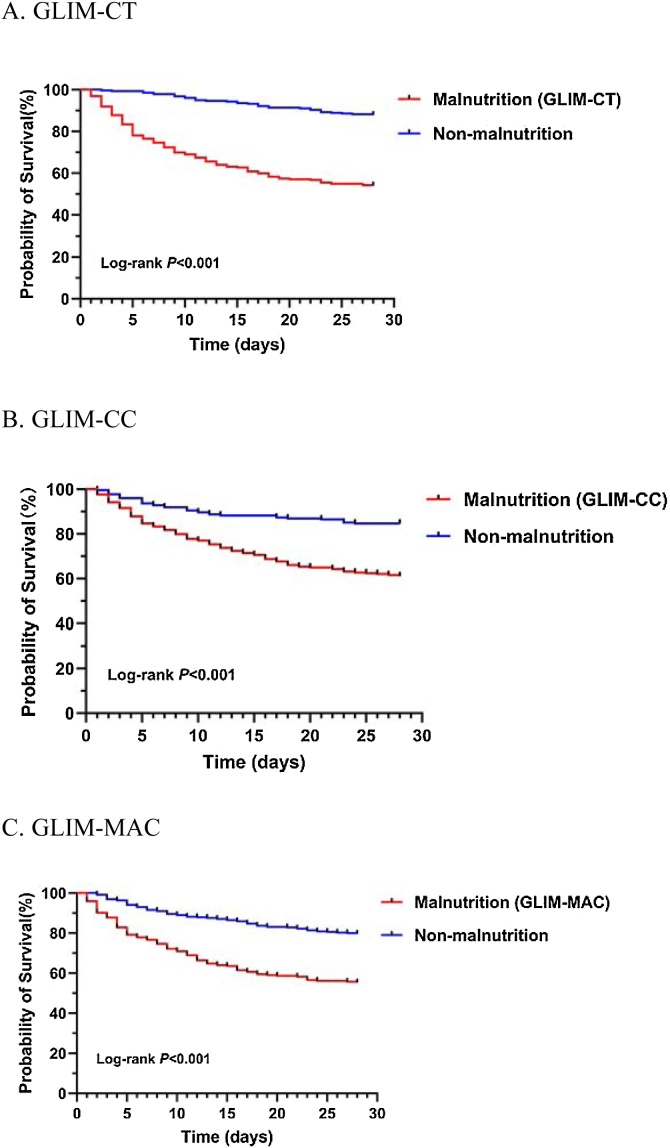
Kaplan–Meier survival curves of malnutrition defined by GLIM criteria.

During follow-up, the 28-day all-cause mortality rate was 30.3% (181 patients died). Univariable Cox regression analysis showed that malnutrition defined according to the three different instruments for muscle mass assessment (GLIM-CT, GLIM-CC, and GLIM-MAC) was independently associated with 28-day mortality (Hazrd Ratio, HR = 4.917, 2.908, and 2.670 respectively; *P* < 0.001). Upon multivariable Cox regression analysis, after adjusting for age, gender, hemoglobin level, albumin concentration, CCI, and SOFA score, GLIM-CT, GLIM-CC, and GLIM-MAC remained independent predictors of 28-day mortality (HR = 4.439, 1.956, and 2.288, respectively; *P* < 0.001, *P* = 0.001, and *P* < 0.001, respectively) (Table 3). Kaplan–Meier survival analysis showed that malnutrition, defined according to the GLIM criteria and three different muscle mass assessment methods was associated with a higher risk of 28-day mortality (Fig. 1).

**Table 3:** Three approaches of GLIM criteria and its association with 28-day all-cause mortality.

	Model 1			Model 2		
	HR	95% CI	*P*	HR	95% CI	*P*
GLIM-CT	4.917	3.384–7.143	<0.001	4.439	3.053–6.454	<0.001
GLIM-CC	2.908	2.002–4.223	<0.001	1.956	1.326–2.885	0.001
GLIM-MAC	2.670	1.982–3.598	<0.001	2.288	1.692–3.093	<0.001

Model 1, unadjusted model; Model 2, adjusted for age, gender, hemoglobin, albumin, CCI and SOFA.

Abbreviations: HR, hazard ratio; CI, confidence interval.

### 3.3 Comparison of the three muscle mass assessment methods

A base model including age, gender, hemoglobin level, albumin concentration, CCI, and SOFA score was constructed, and the C-statistic for the prediction of 28-day mortality was 0.780 (95% CI: 0.741−0.819). Incorporation of GLIM-CT, GLIM-CC, or GLIM-MAC into the base model significantly improved the C-statistic to 0.823 (95% CI: 0.789−0.857), 0.802 (95% CI: 0.765−0.839), and 0.801 (95% CI: 0.764−0.838), respectively (all *P* < 0.001; Table 4). In addition, the NRI and IDI were calculated to compare the predictive abilities of the three instruments. Adding GLIM-CT, GLIM-CC, or GLIM-MAC to the base model significantly improved the model’s predictive ability for 28-day all-cause mortality: the respective NRIs were −0.071 (95% CI: −0.094 to −0.049), −0.023 (95% CI: −0.036 to −0.010), and −0.033 (95% CI: −0.049 to −0.017; all *P* < 0.001). The respective IDIs were −0.799 (95% CI: −0.947 to −0.651), −0.510 (−0.660 to −0.361), and −0.557 (−0.723 to −0.388; all *P* < 0.001).

**Table 4:** Validity of three models of GLIM criteria for 28-day all-cause mortality.

	C-statistic(95%CI)	*P* value	NRI(95%CI)	*P* value	IDI(95%CI)	*P* value
Base Model	0.780 (0.741–0.819)	–	–	–	–	–
Base Model + GLIM-CT	0.823 (0.789–0.857)	<0.001	−0.071 (−0.094, −0.049)	<0.001	−0.799 (−0.947, −0.651)	<0.001
Base Model + GLIM-CC	0.802 (0.765–0.839)	<0.001	−0.023 (−0.036, −0.010)	<0.001	−0.510 (−0.660, −0.361)	<0.001
Base Model + GLIM-MAC	0.801 (0.764–0.838)	<0.001	−0.033 (−0.049, −0.017)	<0.001	−0.557 (−0.723, −0.388)	<0.001
GLIM-CT - GLIM-CC	0.021 (0.001, 0.040)	0.036	−0.049 (−0.066, −0.031)	<0.001	−0.788 (−0.937, −0.640)	<0.001
GLIM-CT - GLIM-MAC	0.021 (0.001, 0.042)	0.042	−0.039 (−0.056, −0.021)	<0.001	−0.518 (−0.687, −0.348)	<0.001
GLIM-CC - GLIM-MAC	0.001 (−0.017, 0.018)	0.950	0.010 (−0.005, 0.025)	0.189	−0.030 (−0.179, 0.118)	0.076

Base Model included: age, gender, hemoglobin, albumin, CCI and SOFA.

Abbreviations: NRI, net reclassification improvement; IDI, integrated discrimination improvement; CI, confidence interval.

For pairwise comparisons between the instruments, the C-statistic of the model including GLIM-CT was higher than those including GLIM-CC and GLIM-MAC (*P* = 0.036 and *P* = 0.042, respectively), and improvements in risk prediction were also confirmed via category-free NRI (*P* < 0.001) and IDI (*P* < 0.001), suggesting that GLIM-CT had better performance than GLIM-CC and GLIM-MAC. The C-statistic did not significantly differ between the models incorporating GLIM-CC or GLIM-MAC (*P* = 0.950), and the NRI and IDI were 0.010 (95% CI: −0.005 to 0.025, *P* = 0.189) and −0.030 (95% CI: −0.179 to 0.118, *P* = 0.076), respectively.

Taken together, the addition of any of the three instruments into the base Cox regression model significantly improved its predictive ability for 28-day mortality in older patients with sepsis in the emergency department; the model including GLIM-CT had the highest C-statistic, and its predictive validity was further confirmed via category-free NRI and IDI.

## 4 Discussion

In the fast-paced emergency department, the identification of malnutrition is crucial yet challenging. In this study, we used three types of muscle mass assessment methods (CT, CC, and MAC) to diagnose malnutrition in older patients with sepsis in an emergency department, according to the GLIM criteria. Malnutrition diagnosed according to each of these methods was independently associated with an adverse clinical outcome. Additionally, a comparison of the predictive validity of the three methods demonstrated that GLIM-CT had the strongest predictive ability for all-cause 28-day mortality. To the best of our knowledge, this is the first study in which the applicability and validity of malnutrition as defined according to the GLIM criteria was explored among older patients in acute care settings in China.

Previous studies have revealed a wide range of prevalence of malnutrition (12%–87%), depending on the heterogeneity of the study population, diagnostic criteria, and clinical setting [23]. To date, no gold-standard assessment tool for malnutrition has been universally endorsed [24]. The GLIM criteria proposed in 2018 are becoming widely accepted diagnostic standards. Reduced muscle mass is an important component of the phenotypic criteria, but currently, there are no clearly defined cut-off values in GLIM criteria [11]. The guidance for assessment of the muscle mass phenotypic criterion for GLIM recommended thresholds for CC [17], but there were no cut-off values for CT and MAC. In our study, we utilized the cut-off values for reduced muscle mass as established in previous relevant studies and literature that were specifically tailored to the Asian population. The prevalence of malnutrition defined according to the GLIM criteria ranged from 40.8% (GLIM-MAC) to 63.0% (GLIM-CC). The concordance of all three methods was relatively good, the highest being between GLIM-CT and GLIM-MAC (Cohen’s kappa coefficient = 0.706, *P* < 0.001). In a previous study of older Chinese inpatients, the incidence of GLIM-defined malnutrition was 27.8% according to various tools [25], which is lower than that in our study. This discrepancy may be attributed to the specific characteristics of the study population (i.e., patients with sepsis) and the clinical setting (i.e., the emergency department).

Muscle mass assessment is an essential component of the GLIM criteria, and previous studies have indicated that it enhances the overall performance of the GLIM criteria [26,27]. However, skeletal muscle mass is less commonly assessed in critically ill patients, especially in emergency settings [24,28]. Barazzoni et al. reviewed various muscle assessment tools and noted that DXA and BIA are not widely used in clinical practice [17,29,30]. Anthropometric measures, such as CC and MAC, as well as functional assessments, such as HGS, may serve as alternatives, albeit with lower accuracy. Currently, CT is routinely performed for patients with sepsis to identify the source of infection or infectious state, and secondary analysis of such images can be used to determine skeletal muscle mass without additional costs or radiation exposure [31]. Therefore, in this study, we employed the latter three muscle assessment modalities to evaluate malnutrition status, defined according to the GLIM criteria, in older patients with sepsis. A study comparable to the present one was conducted by Liang et al. [19]; however, they focused on in-patients with heart failure. They analyzed GLIM-CC, GLIM-MAC, and GLIM-physical examination. However, in our study, the GLIM-CT model had the highest predictive value for 28-day mortality, and when imaging technologies are not available, GLIM-CC and GLIM-MAC may serve as adequate alternatives to diagnose malnutrition and predict the prognosis. Another study on older patients undergoing surgery revealed that the GLIM-CC had good performance and validity in the prediction of in-hospital mortality [32]. Therefore, multiple tools for the assessment of muscle mass reduction can be incorporated into malnutrition evaluation. Emergency physicians may select appropriate modalities based on the clinical context, patient characteristics, and resource availability.

Our study has certain limitations. First, it was a single-center study conducted in the emergency department, and only older patients with sepsis who underwent abdominal CT were included, which might have led to selection bias and must be considered when our results are interpreted. Second, patients with geriatric syndromes, such as frailty, polypharmacy, and dysphagia, were not included in the analysis, all of which may affect nutritional status and clinical outcomes. Third, as we did not incorporate the GLIM thresholds for muscle mass reduction as measured via DXA or BIA recommended in GLIM criteria, we were unable to estimate the accuracy of the three methods used in this study for the assessment of malnutrition. Meanwhile, we did not measure the concurrent validity of the GLIM criteria with other nutritional assessments, such as the subjective global assessment. Finally, the severity of malnutrition was not assessed because no cutoffs for the stratification of moderate or severe reductions in muscle mass have been identified. Therefore, large-scale, multi-center studies are needed to determine cut-offs for different techniques and validate different assessment tools in clinical settings.

## 5 Conclusion

Malnutrition, defined via any of the three GLIM-related measurement methods, was prevalent among older patients with sepsis in the emergency department and was associated with 28-day mortality. GLIM-CT, using L3 SMI for the assessment of muscle mass reduction, had the best predictive validity. This study provides novel evidence for the application and validity of the GLIM criteria by emergency physicians among older patients with sepsis. Furthermore, it may be of use for risk stratification, clinical decision-making, and evidence-based allocation of resources in clinical practice.

## CRediT authorship contribution statement

Na Shang was responsible for proposing the study protocol and data organization, as well as completing the drafting of the article. Qiujing Li participated in data analysis and the revision of the paper. Haijiang Zhou and Xiangqun Zhang were in charge of providing resource support and verifying the research results. Guo Shubin and Mei Xue contributed to the proofreading, reviewing, and providing academic guidance.

## Ethics approval and consent to participate

This study was approved by the institutional review board of Beijing Chao-Yang Hospital affiliated to Capital Medical University (approval number: 2022-ke-430, August 1, 2022) and conducted in accordance with the amended Declaration of Helsinki. This study was registered at www.chictr.org.cn (registration number ChiCTR2300070377).

## Declaration of Generative AI and AI-assisted technologies in the writing process

I have not used any AI at all.

## Funding

This work was supported by the Capital’s Funds for Health Improvement and Research [grant numbers 2024-2-2034].

## Availability of data and materials

The data that support the findings of this study are available from the corresponding author upon reasonable request.

## Declaration of competing interest

No potential conflicts of interest related to this article are reported.

## Footnotes

1 BIA, bioelectrical impedance analysis; BMI, body mass index; CC, calf circumference; CCI, Charlson Comorbidity Index; CI, confidence interval; CT, computed tomography; DXA, dual-energy absorptiometry; ESPEN, European Society for Parenteral and Enteral Nutrition; GLIM, Global Leadership Initiative on Malnutrition; HGS, hand-grip strength; HR, hazard ratio; IDI, integrated discrimination improvement; L3, third lumbar vertebra; MAC, mid-upper-arm circumference MRI, magnetic resonance imaging; NRI, net reclassification improvement; SMA, skeletal mass area; SMI, skeletal mass index; SOFA, sequential organ failure assessment
